# Temperature and Frequency Dependence of the Dynamic Viscoelastic Properties of Silicone Rubber

**DOI:** 10.3390/polym15143005

**Published:** 2023-07-10

**Authors:** Xiu Liu, Dingxiang Zhu, Jianguo Lin, Yongjun Zhang

**Affiliations:** 1 School of Mechanical Engineering and Mechanics, Xiangtan University, Xiangtan 411105, China; liux@xtu.edu.cn (X.L.); dxzhu98@gmail.com (D.Z.); 2 School of Materials Science and Engineering, Xiangtan University, Xiangtan 411105, China; 3 School of Architectural Engineering, Hunan Institute of Engineering, Xiangtan 411104, China

**Keywords:** time–temperature equivalence principle, dynamic viscoelastic properties, master curve, WLF equation, fractional-order derivative viscoelastic model

## Abstract

Temperature–frequency sweep tests were performed on silicone rubber to investigate the dynamic viscoelastic properties. The test results show that the viscoelasticity of silicone rubber presents significant temperature dependence and frequency dependence. The dynamic viscoelastic test curves at different temperatures can be shifted along the logarithmic frequency coordinate axis to construct smooth master curves at the reference temperature of 20 °C, covering a frequency range of 10 decades, which indicates thermorheological simplicity on a macro level and frequency temperature equivalence of the silicone rubber material in the experimental temperature range. The van Gurp–Palmen plot and Cole–Cole plot for the test data at various temperatures merge into a common curve, which further validates thermorheological simplicity. The temperature dependent shift factors of silicone rubber material were well characterized by the Williams–Landel–Ferry equation. Moreover, the fractional-order differential Kelvin (FDK) model, the fractional-order differential Zener (FDZ) model, and the improved fractional-order differential Zener (iFDZ) model were used to model the asymmetric loss factor master curve. The result shows that the iFDZ model is in good agreement with the test results, indicating that this model is suitable for describing the asymmetry of dynamic viscoelastic properties of silicone rubber.

## 1. Introduction

Rubber has unique physical and mechanical properties such as hyperelasticity, viscoelasticity, wear resistance, and insulation. It is widely used in national production and the defense industry, e.g., in automotive tire manufacturing, rail transportation, and underground protection projects. A material is viscoelastic when it exhibits a combination of both elasticity and viscosity. Rubber is one of the most common viscoelastic materials. The dynamic viscoelastic properties are essential for the structural design and life assessment of rubber.

Compared with general purpose rubbers, such as ethylene propylene diene monomer [1] and polymerized styrene butadiene rubber [2], silicone rubber has excellent high- and low-temperature resistance [3], chemical stability, oxygen aging resistance, gas permeability, electrical insulation, resilience and flexibility [4], oil resistance, solvent resistance, and radiation resistance. It is widely used in aerospace, electronics appliances, chemical instrumentation, machinery manufacturing, construction, daily life, and other fields. In addition, silicone rubber is widely used in engineering materials because of its excellent properties of vibration and shock dampening [1]. Therefore, the study of the dynamic mechanical properties of silicone rubber is of great practical significance for fatigue failure analysis and life assessment. The main research in this regard examines the significant elasticity of rubber. Many constitutive models have been proposed to display the complicated behavior of silicone rubber, which includes hyperelasticity [5], viscoelasticity [6], and visco-hyperelastic behavior [7].

It was found that the mechanical properties of rubber are affected by temperature and time. There is a certain equivalence between the two, which is referred to as the time–temperature equivalence principle, also known as the time–temperature superposition principle (TTSP), and with the change of temperature, the rubber material can also present three different mechanical forms, which are viscous flow state, rubber state, and glass state. The TTSP is an essential tool for studying temperature–frequency effects of viscoelastic materials. Based on the TTSP, dynamic frequency sweep test data of thermorheological simple materials at different temperatures can be shifted along the logarithmic frequency axis to obtain a series of master curves at a reference temperature. These can be used to predict the linear viscoelastic mechanical response of the material on a broader time/frequency domain, which is widely used in the analysis of the mechanical behavior of viscoelastic materials.

Most of the research on time–temperature equivalence is focused on the determination of the parameters of the Williams–Landel–Ferry (WLF) equation and its application extension, the error between the WLF equation’s prediction and the actual results, the prediction of new material properties, and the validation of dynamic mechanical models [8,9]. Paulo et al. [10] investigated the rheological behavior of tire rubber at a constant shear rate using the time–temperature equivalence principle and obtained well-fitted results. Lin et al. [11] applied the WLF equation to establish the relationship between temperature and reversible phases for the shape memory of linear ether polyurethanes. Jacek et al. [12] discussed the influential nature of the parameters in the WLF equation at the molecular level. Zhang et al. [13] proposed the frequency spectrum–temperature spectrum mirror relationship for viscoelastic materials and derived a six-parameter fractional-order model for the temperature spectrum which was validated by dynamic mechanical analysis (DMA) tests. Hu et al. [14] studied the Payne effect and hysteresis loss of carbon black-filled rubber at different temperatures and proposed a method for accelerated assessment of the Payne effect at arbitrary temperatures that can be based on fewer test data according to the WLF equation. Liang et al. [15] proposed a fractional-order differential principal structure model in order to accurately describe the linear viscoelastic properties of asphalt and asphalt mastic—the generalized fractional-order differential Zener model. Luo et al. [16] determined the S-N curve in the traditional sense with the maximum principal strain as the fatigue parameter, established the relationship between the steady-state temperature rise and the maximum principal strain, and proved that the steady-state temperature rise could effectively evaluate the fatigue life of rubber members.

The current research on viscoelastic materials’ dynamic mechanical frequency effects is mainly based on the principal structure equation [17]. The dynamic mechanical properties, such as storage modulus and loss factor containing frequency variables, are obtained by extrapolating them from the real domain to the complex domain and separating their imaginary parts through Laplace transform [18]. Nutting et al. [19] first developed a fractional exponential model to describe the stress relaxation phenomenon of rubber. Subsequently, Muhammad et al. [20] and Bosworth et al. [21] first proposed a fractional-order derivative model for viscoelastic media, after which scholars at home and abroad conducted a great deal of research on the fractional-order model of viscoelasticity and arrived at many useful conclusions [22,23]. Tang et al. [24] proposed a five-parameter fractional derivative rubber vibration isolator constitution model, extrapolated to the frequency domain, and identified the parameters. Li et al. [25] established a fractional-order viscoelastic oscillator model considering shape parameters and applied it to the dynamic analysis of viscoelastic suspensions for tracked vehicles. Wharmby et al. [26] established a modified Maxwell instanton equation for viscoelastic materials based on fractional-order derivatives and obtained its frequency response function by pull-type transformation. Cao et al. [27] proposed a fractional-order weighted distribution parameter Maxwell model and obtained its time domain response by pull-type inversion transformation.

Few studies have examined the time–temperature equivalence of silicone rubber, the relationship between the WLF parameters and their variation, and the viscoelastic constitutive description over a wide frequency domain, despite the fact that the rheological behavior of viscoelastic materials and their constitutive models have received extensive attention. In this study, we focus on the temperature-dependent dynamic rheological behavior of silicone rubber and the appropriate fractional-order constitutive model. We used the Gabo Eplexor 500 N dynamic thermodynamic analyzer to perform temperature–frequency sweep tests (−35 °C~60 °C) on silicone rubber to obtain the test curves of storage modulus *E*′, loss modulus *E*″, and loss factor tan *δ* at different temperatures. Based on the TTSP, the dynamic viscoelastic master curves were constructed at the reference temperature of 20 °C. The WLF equation was applied to fit the temperature shift factors nonlinearly, and the parameters of the WLF equation were discussed. The van Gurp–Palmen plot and Cole–Cole plot for the test data at various temperatures were used to verify the thermal rheological properties of silicone rubber. Moreover, the dynamic rheological behavior of silicone rubber was characterized using the fractional-order differential Zener model and the improved fractional-order differential Zener model.

## 2. Theory

### 2.1. Time–Temperature Superposition Principle

The viscoelastic mechanical behavior of a viscoelastic material can be measured both at a lower temperature and longer time (lower frequency) action and presented at a higher temperature and shorter time (higher frequency) action. The effect of temperature is the same as the effect of action time, and the viscoelastic material is said to be a thermorheological simple material on a macro level when the material satisfies the TTSP [28]. According to the TTSP, the isothermal curve can be realized to be shifted down to the reference temperature, extending the range of the frequency spectrum at the reference temperature with the relation:

(1) $$Y\left(f,T\right)=Y\left(\frac{f_{r}}{\phi_{T}},T_{r}\right)$$

where *Y* is the dynamic property of the viscoelastic material (e.g., storage modulus *E*′, loss modulus *E*′’, or loss factor tan *δ*); *f* and *f*_r_ are the load frequency and scaling frequency, respectively; *T* and *T*_r_ are environmental temperature and reference temperature, respectively; and *ϕ*_T_ is frequency conversion factor. Then:

(2) $$f=\frac{f_{r}}{\phi_{T}}$$

Furthermore:

(3) $$\phi_{T}=\frac{f_{r}}{f}$$

The frequency-transformation factor *ϕ*_T_ is the amount of translation to achieve the frequency spectrum of dynamic mechanical properties at temperature *T* to the reference *T*_r_. The master curve is the broadband performance of the material at the reference temperature *T*_r_, thus extending the predictive capability of the dynamic properties at the reference temperature.

### 2.2. The WLF Equation

Using the frequency conversion factor *ϕ*_T_ as a function of temperature, Williams, Landel, and Ferry found that near the glass transition temperature, for almost all amorphous polymers, the relationship between the shift factor log*ϕ*_T_ and (*T* − *T*_r_) satisfies the equation:

(4) $$\log\phi_{T}=\frac{-C_{1}\left(T-T_{r}\right)}{C_{2}+\left(T-T_{r}\right)}$$

This is the WLF equation, where *C*_1_ and *C*_2_ are the material parameters.

Using the conversion factor *ϕ*_T_, the conversion frequencies *w*_r_ at different temperatures can be obtained to achieve the correlation of the dynamic viscoelasticity at different temperatures:

(5) $$\omega_{r}=\omega \phi_{T}$$

where *w* is the angular frequency.

### 2.3. Fractional-Order Derivative Viscoelastic Model

Viscoelastic materials have mechanical properties between elasticity and viscosity and can, therefore, be simulated by a viscoelastic model that combines elastic and viscous components. The constitutive models describing the mechanical behavior of viscoelasticity can be divided into two types: differential and integral models. Among these, the differential constitutive model is more common. The differential constitutive model can be divided into the integer-order differential constitutive model and fractional-order differential constitutive model.

**Figure 1:**
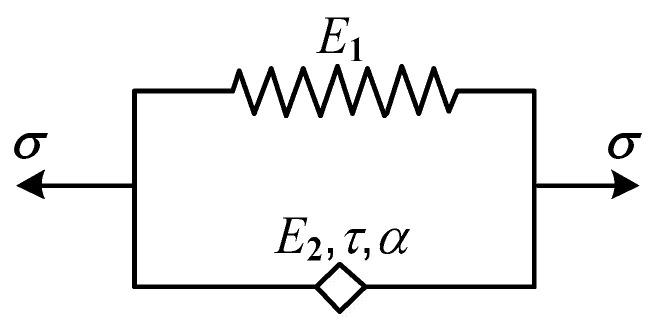
Fractional-order differential Kelvin–Voigt model.

The fractional-order differential Kelvin–Voigt model can be obtained by replacing the viscous pot in the classical integer-order Kelvin–Voigt model with the Koeller pot [29], as shown in Figure 1.

The present constitutive equation is obtained by:

(6) $$\sigma \left(t\right)=\left(E_{1}+E_{2}\tau^{\alpha }D^{\alpha }\right)\varepsilon \left(t\right)$$

where *E*_1_ is the modulus of the spring element; *E*_2_, *τ*, and *α* are the modulus, average relaxation time, and fractional order of spring pot element, respectively; 0 < *α* < 1; D represents the Riemann Liouville fractional derivative operator; and D*^α^* is defined as [30]:

(7) $$D^{\alpha }f\left(t\right)=\frac{1}{\Gamma \left(1-\alpha \right)}\frac{d}{dt}\int_{0}^{t}{\left(t-\tau \right)}^{-\alpha }f\left(\tau \right)d\tau ,f\left(0\right)=0$$

In the formula, Γ(x) is the Eulerian gamma function.

To describe the dynamic viscoelastic properties of the material, the Fourier transform of Equation (6) yields the complex modulus of the fractional-order differential Kelvin–Voigt model:

(8) $$E^{*}\left(i\omega \right)=E_{1}+E_{2}\tau^{\alpha }{\left(i\omega \right)}^{\alpha }$$

Substituting $i^{\alpha }=\cos\left(\alpha \pi /2\right)+i\sin\left(\alpha \pi /2\right)$ into Equation (8) and separating the real and imaginary parts yields the storage modulus *E*′(*w*), loss modulus *E*″(*w*), and loss factor tan *δ*:

(9) $$E^{\prime }\left(\omega \right)=E_{1}+E_{2}{\left(\tau \omega \right)}^{\alpha }\cos\left(\alpha \pi /2\right)$$

(10) $$E^{\prime \prime }\left(\omega \right)=E_{2}{\left(\tau \omega \right)}^{\alpha }\sin\left(\alpha \pi /2\right)$$

(11) $$\tan\delta =\frac{E_{2}{\left(\tau \omega \right)}^{\alpha }\sin\left(\alpha \pi /2\right)}{E_{1}+E_{2}{\left(\tau \omega \right)}^{\alpha }\cos\left(\alpha \pi /2\right)}$$

**Figure 2:**
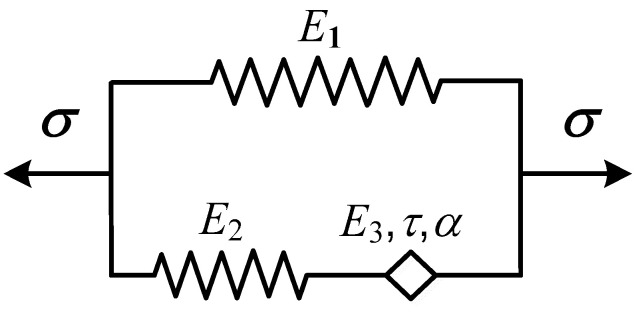
Fractional-order differential Zener model.

The fractional-order differential Zener model can be obtained by replacing the viscous pot in the classical integer-order Zener model with the Koeller pot, as shown in Figure 2.

The present constitutive equation can be expressed by:

(12) $$\sigma \left(t\right)=\left(E_{1}+\frac{E_{2}E_{3}\tau^{\alpha }D^{\alpha }}{E_{2}+E_{3}\tau^{\alpha }D^{\alpha }}\right)\varepsilon \left(t\right)$$

In the same way, it can be deduced that the storage modulus *E*′(*w*), loss modulus *E*″(*w*), and loss factor tan *δ*:

(13) $$E^{\prime }\left(\omega \right)=\frac{E_{1}E_{2}{}^{2}+\left(2E_{1}+E_{2}\right)E_{2}E_{3}{\left(\omega \tau \right)}^{\alpha }\cos\left(\alpha \pi /2\right)+\left(E_{1}+E_{2}\right)E_{3}{}^{2}{\left(\omega \tau \right)}^{2\alpha }}{E_{2}{}^{2}+2E_{2}E_{3}{\left(\omega \tau \right)}^{\alpha }\cos\left(\alpha \pi /2\right)+E_{3}{}^{2}{\left(\omega \tau \right)}^{2\alpha }}$$

(14) $$E^{\prime \prime }\left(\omega \right)=\frac{E_{2}{}^{2}E_{3}{\left(\omega \tau \right)}^{\alpha }\sin\left(\alpha \pi /2\right)}{E_{2}{}^{2}+2E_{2}E_{3}{\left(\omega \tau \right)}^{\alpha }\cos\left(\alpha \pi /2\right)+E_{3}{}^{2}{\left(\omega \tau \right)}^{2\alpha }}$$

(15) $$\tan\delta =\frac{{\left(\omega \tau \right)}^{\alpha }\sin\left(\frac{\alpha \pi }{2}\right)}{\frac{E_{1}}{E_{3}}+\left(1+\frac{2E_{1}}{E_{2}}\right){\left(\omega \tau \right)}^{\alpha }\cos\left(\frac{\alpha \pi }{2}\right)+\left(1+\frac{E_{1}}{E_{2}}\right)\frac{E_{3}}{E_{2}}{\left(\omega \tau \right)}^{2\alpha }}$$

**Figure 3:**
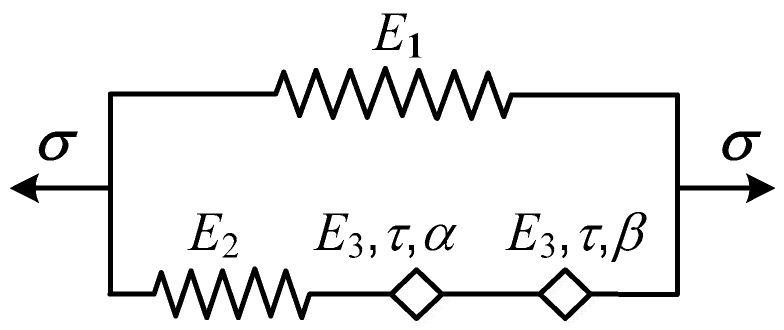
Improved fractional-order differential Zener model.

The fractional-order differential Zener model describes the instantaneous elasticity of a solid with steady-state asymptotic values. It is usually used to describe the dynamic mechanical properties of viscoelastic materials over a wide frequency range. The Koeller pots of the Zener model can be extended into two pots in series to obtain a kind of improved fractional-order differential Zener model [31], as shown in Figure 3. To reduce the number of model parameters, the elastic modulus and mean relaxation time of the two pots are set to be the same, denoted by *E*_3_ and *τ*, and the fractional-order differentiation is different, denoted by *α* and *β*.

After simple derivation, the constitutive equation of the improved fractional-order differential Zener model can be obtained:

(16) $$\sigma \left(t\right)=\left(E_{1}+\frac{1}{\frac{1}{E_{2}}+\frac{1}{E_{3}\tau^{\alpha }D^{\alpha }}+\frac{1}{E_{3}\tau^{\beta }D^{\beta }}}\right)\varepsilon \left(t\right)$$

Similarly, we can deduce the storage modulus *E*′(*w*), loss modulus *E*″(*w*), and loss factor tan *δ*:

(17) $$E^{\prime }(\omega )=\frac{E_{1}E_{2}{}^{2}\lambda_{1}+\left(E_{1}+E_{2}\right)E_{3}^{2}{\left(\omega \tau \right)}^{2\left(\alpha +\beta \right)}+\left(2E_{1}+E_{2}\right)E_{2}E_{3}{\left(\omega \tau \right)}^{\alpha +\beta }\lambda_{2}}{E_{3}{}^{2}{\left(\omega \tau \right)}^{2\left(\alpha +\beta \right)}+E_{2}{}^{2}\lambda_{1}+2E_{2}E_{3}{\left(\omega \tau \right)}^{\alpha +\beta }\lambda_{2}}$$

(18) $$E^{\prime \prime }(\omega )=\frac{E_{2}^{2}E_{3}{\left(\omega \tau \right)}^{\alpha +\beta }\lambda_{3}}{{\left(\omega \tau \right)}^{2(\alpha +\beta )}E_{3}{}^{2}+E_{2}{}^{2}\lambda_{1}+2E_{2}E_{3}{\left(\omega \tau \right)}^{\alpha +\beta }\lambda_{2}}$$

(19) $$\tan\delta =\frac{{\left(\omega \tau \right)}^{\alpha +\beta }\lambda_{3}}{\frac{E_{1}}{E_{3}}\lambda_{1}+\left(1+\frac{E_{1}}{E_{2}}\right)\frac{E_{3}}{E_{2}}{\left(\omega \tau \right)}^{2(\alpha +\beta )}+\left(1+2\frac{E_{1}}{E_{2}}\right){\left(\omega \tau \right)}^{\alpha +\beta }\lambda_{2}}$$

Among them: $\lambda_{1}={\left(\omega \tau \right)}^{2\alpha }+{\left(\omega \tau \right)}^{2\beta }+2{\left(\omega \tau \right)}^{\alpha +\beta }\cos\left(\frac{\alpha -\beta }{2}\pi \right)$, $\lambda_{2}={\left(\omega \tau \right)}^{\beta }\cos\left(\frac{\alpha \pi }{2}\right)+{\left(\omega \tau \right)}^{\alpha }\cos\left(\frac{\beta \pi }{2}\right)$, $\lambda_{3}={\left(\omega \tau \right)}^{\beta }\sin\left(\frac{\alpha \pi }{2}\right)+{\left(\omega \tau \right)}^{\alpha }\sin\left(\frac{\beta \pi }{2}\right)$.

## 3. Experimental Section

**Figure 4:**
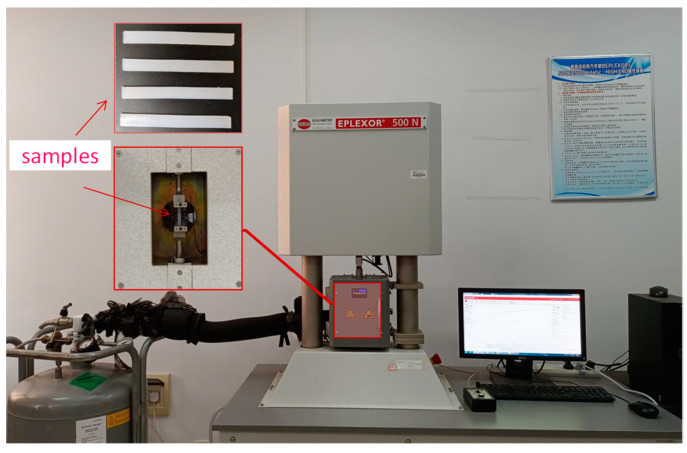
Dynamic mechanical analysis test equipment.

The test material was semitransparent silicone rubber sheet, obtained through vulcanization and extrusion, with a ShorE′s hardness of around 65 degrees, from Care Measurement and Control Test System (Tianjin) company. The thin rectangular strip used for DMA testing was 50 mm long, 5 mm wide, and 2 mm thick. The DMA test equipment was a Gabo Eplexor 500 N dynamic thermodynamic analyzer, as shown in Figure 4. In order to avoid the Mullins effect on the deformation cycle of the material, mechanical pretreatment was performed on all specimens. A sinusoidal strain was applied to the silicon rubber specimens with a prestrain of 0.8% and a superimposed dynamic strain amplitude of 0.2% which was kept in the linear viscoelastic region of the measurement.

Temperature–frequency sweep tests were carried out on the samples of silicon rubber in the 0.1 Hz to 70 Hz range and repeated at different temperatures from −35 °C to 60 °C with 5 K intervals. During these sweep measurements, the stress response to strain excitation was automatically recorded and *E*′, *E*″, and tan *δ* were calculated from these measurements.

## 4. Results and Discussion

### 4.1. Master Curve Analysis

**Figure 5:**
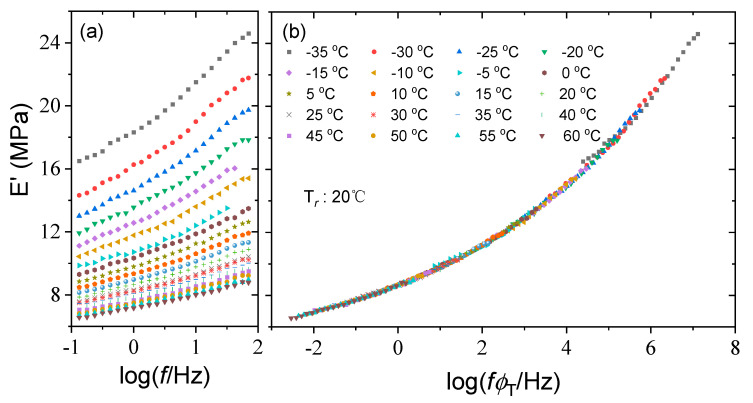
Test curves of storage modulus of silicone rubber at various temperatures (**a**), and the corresponding master curve at a reference temperature of 20 °C (**b**).

The curves of storage modulus versus loading frequency for silicone rubber at different temperatures are shown in Figure 5a. The storage modulus E′ represents the energy stored in the material during deformation due to elastic deformation. As shown in the figure, the value of the storage modulus E′ of the silicone rubber specimen varies from 0.13 to 24.59 MPa with temperature and frequency. The variation law of the storage modulus E′ of the material with temperature and frequency is consistent with the results of Sawai [32], Placet [33], and others. In general, for viscoelastic solid materials, the storage modulus E′ increases with the increase of test frequency [34]. As the test frequency increases, the molecular chain segment motion of the silicone rubber specimen lags behind the change in external force and the internal consumption decreases. The material becomes more rigid and exhibits the mechanical properties of the glassy state, which is manifested macroscopically as an increase in the value of the storage modulus E′.

According to the TTSP, the frequency conversion factor can be used to shift the test frequency spectrum at multiple sets of temperatures to obtain the master curve at the reference temperature, thus enabling the prediction of dynamic mechanical properties in the high/low-frequency band that the test equipment cannot cover. Before conducting the time–temperature equivalence analysis, the reference temperature that constitutes the master curve must be selected. In this experiment, 20 °C was used as the reference temperature for the storage modulus master curve. In the logarithmic frequency coordinate, the measured storage modulus test curve at the reference temperature does not shift. However, the test curve above or below this temperature is shifted horizontally by CFS algorithm [35] to the left or right along the frequency axis so that all the curves are superimposed on each other and connected. Then, the smooth master curve of storage modulus is constructed only through horizontal shift, without vertical shift, as shown in Figure 5b, and the corresponding frequency conversion factors are shown in Table 1. It is worth mentioning that the shifting error caused by CFS algorithm is at least 10–50 times smaller than the underlying experimental error, which indicates that the master curve constructed is accurate enough.

**Figure 6:**
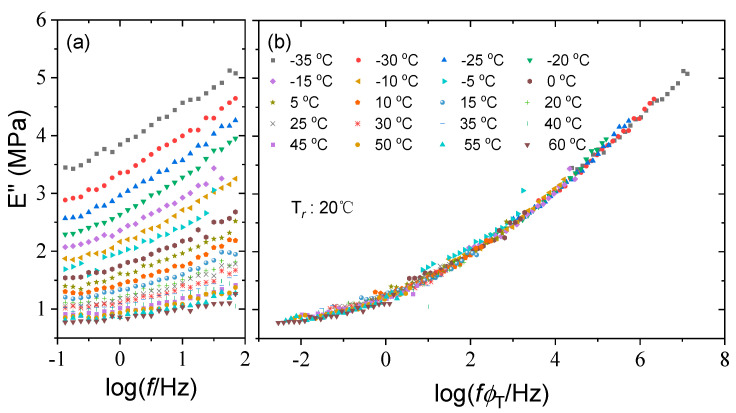
Test curves of loss modulus of silicone rubber at various temperatures (**a**) and the corresponding master curve at a reference temperature of 20 °C (**b**).

**Figure 7:**
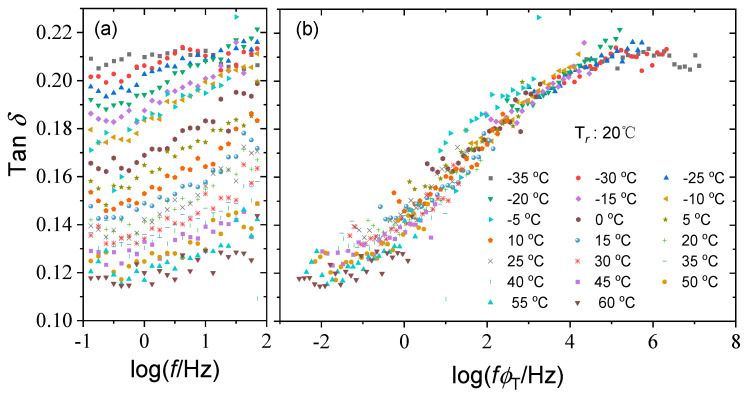
Test curves of loss factor of silicone rubber at various temperatures (**a**) and the corresponding master curve at the reference temperature of 20 °C (**b**).

The acquired shift factors of the storage modulus in Table 1 are used to construct the master curves for the loss modulus and loss factor at a reference temperature of 20 °C. The resulting master curves are quite smooth and cover a frequency range of 10 decades from 10^−2^~10^8^ Hz., as seen in Figure 6 and Figure 7, revealing the thermorheological simplicity of silicone rubber. This indicates that the time temperature equivalence principle is applicable to viscoelastic mechanical behavior of silicone rubber within the test temperature range and presents an excellent accelerated characterization to expand the frequency range.

**Figure 8:**
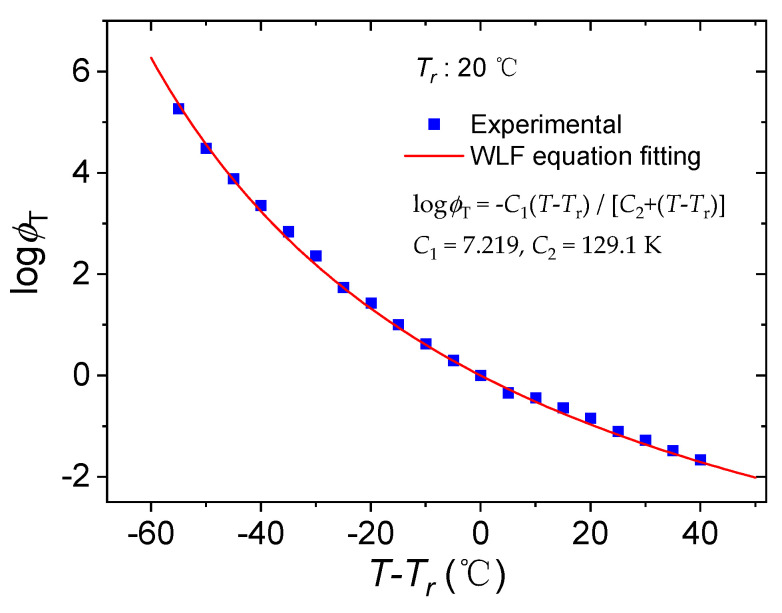
Experimentally determined temperature shift factors of silicon rubber and the fitted curve by WLF equation.

The temperature shift factor, which represents the movement of each viscoelastic unit and the ratio of the material’s relaxation time at a given temperature to the relaxation time at the reference temperature, is used to create the master curve by characterizing the horizontal displacement of each experimental curve in the frequency coordinate. The temperature shift factor *ϕ*_T_ is a function of temperature, and the WLF Equation (4) is often used to analyze the *ϕ*_T_ of the master curve of the dynamic viscoelastic parameters of the material in addition to obtaining the value of *ϕ*_T_ by means of a computation of the experimental data. Figure 8 shows the temperature shift factor *ϕ*_T_ and the fitted curve of the WLF equation for the master curves of dynamic modulus of silicone rubber at the reference temperature of 20 °C. As can be seen from the figure, the WLF equation is in good agreement with the temperature shift factors obtained from the experiment. The fitting determination coefficient is 0.9982.

As can be seen from Figure 8, as the difference between the test temperature and the reference temperature increases, i.e., (*T* − *T*_r_), the temperature shift factor *ϕ*_T_ decreases, and when (*T* − *T*_r_) is less than 0 °C, *ϕ*_T_ is more obvious; when (*T* − *T*_r_) is greater than 0 °C, *ϕ*_T_ declines slowly. This indicates that the closer the test temperature is to the reference temperature in constituting the master curve, the shorter the distance for the test curve at that test temperature to make shifts, indicating a smaller *ϕ*_T_. When the test temperature is higher than the reference temperature, the logarithmic temperature shift factor log*ϕ*_T_ is negative, indicating that the relaxation time of the material molecular motion shortens as the temperature increases. This corresponds to a higher frequency of the alternating load. The fitted curve of the WLF equation is well approximate to the test data and can predict the temperature shift factor *ϕ*_T_ to a certain extent.

The WLF equation to the experimental data at the reference temperature of 20 °C yields *C*_1_ = 7.219, *C*_2_ = 129.1 K, from which it can be seen that the product of the obtained material parameters *C*_1_ and *C*_2_ matches the empirical value of the product of *C*_1_ and *C*_2_ for polymeric materials, which is approximately equal to 900 K [36].

**Figure 9:**
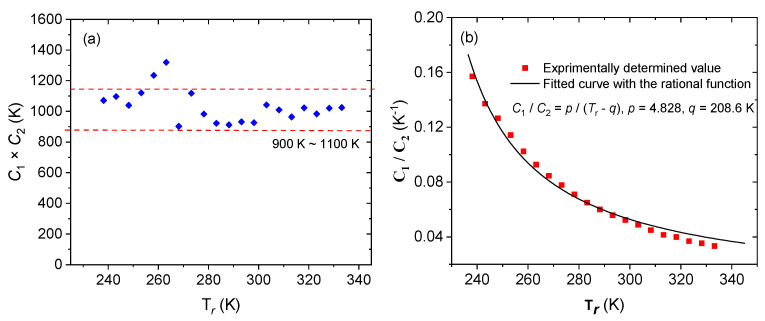
Variation of the product of *C*_1_ and *C*_2_ with reference temperature (**a**), and the ratio of *C*_1_ to *C*_2_ with reference temperature (**b**).

To further investigate the law of the WLF equation, *C*_1_ and *C*_2_ at different reference temperatures were obtained by the same method, as shown in Table 2 and Figure 9. From Figure 9a, one can see that the product of material parameters *C*_1_ and *C*_2_ is stable between 900 K and 1100 K, and from Figure 9b, one can observe that *C*_1_/*C*_2_ shows a rational function relationship with reference temperature:

(20) $$C_{1}/C_{2}=p/(T_{r}-q)$$

To further verify the thermorheological simplicity of silicone rubber, van Gurp–Palmen plots and the Cole–Cole plot are provided. Van Gurp and Palmen [37] proposed a method to verify the time–temperature equivalence principle by plotting the hysteresis phase δ against the absolute value of the complex modulus |E^*^| and found that if it holds, the frequency curves of the isotherms merge into a typical curve. This plotting method avoids shifting the data along the frequency axis and results in a temperature-independent curve. Thus, any breakdown of the time–temperature equivalence principle can be easily seen from the van Gurp–Palmen diagram. This has proven to be a practical tool for detecting the simplicity of thermorheology. By graphing *E*″ vs. *E*′, the Cole–Cole plot describes the time-dependent dynamic viscoelastic moduli. While *E*″ and *E*′ are depicted on linear axes in the standard Cole–Cole plot, logarithmic axes are used in the modified Cole–Cole plot.

**Figure 10:**
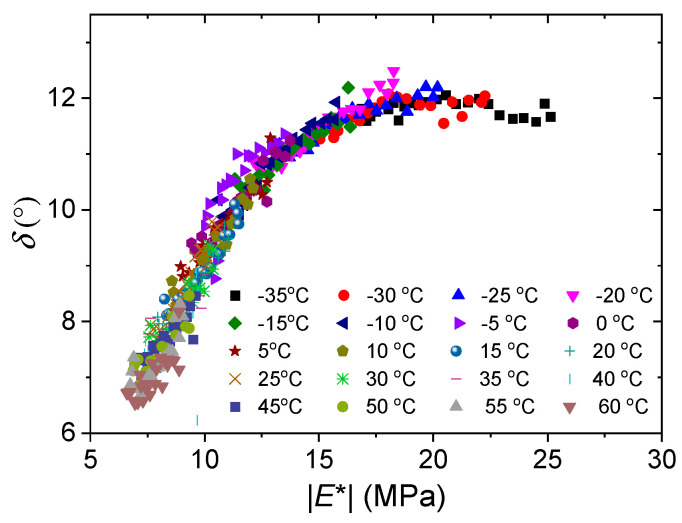
Van Gurp–Palmen plot of phase lag δ versus absolute value of complex modulus |E^*^| at various temperatures.

**Figure 11:**
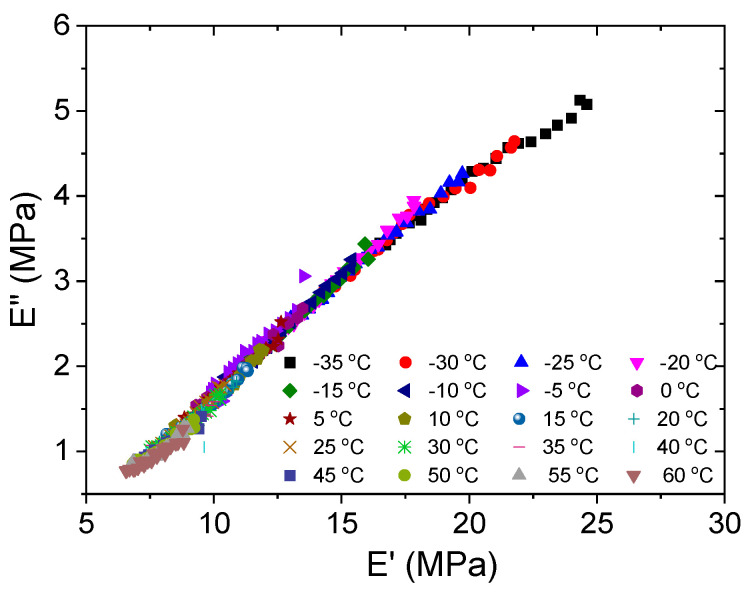
Cole–Cole plot of loss modulus *E*″ versus storage modulus *E*′ at various temperatures.

Figure 10 and Figure 11 show the van Gurp–Palmen plot and the Cole–Cole plot of the test data. It can be seen from both plots that no processing of the data is required to superimpose the isothermal sweep curves into a single curve. Thus, a strong case can be made that silicone rubber is thermorheologically simple from the macroscopic view, which means it meets the equivalence of time and temperature in the experimental temperature range. It is worth noting that the single Cole–Cole curve and the van Gurp–Palmen curve indicate that the microstructure of the silicone rubber used in the test hardly changes with temperature in the experimental range. Perhaps because silicone rubber is an excellent high-temperature- and low-temperature-resistant material, its microstructure does not undergo significant changes with temperature.

### 4.2. Application Analysis of Fractional Derivative Model

Both the differential rheological model and integral rheological model are widely used to describe the viscoelasticity mechanical behavior of rheological material. The integral rheological model is generally used for creep and stress relaxation, which is convenient to describe the load history-dependent viscoelasticity behavior. However, the differential rheological model is more graphical for its visual model diagram than the integral model and can be more conveniently applied to the field of dynamic viscoelasticity for it is easy to perform Fourier transform from time domain to frequency domain.

**Figure 12:**
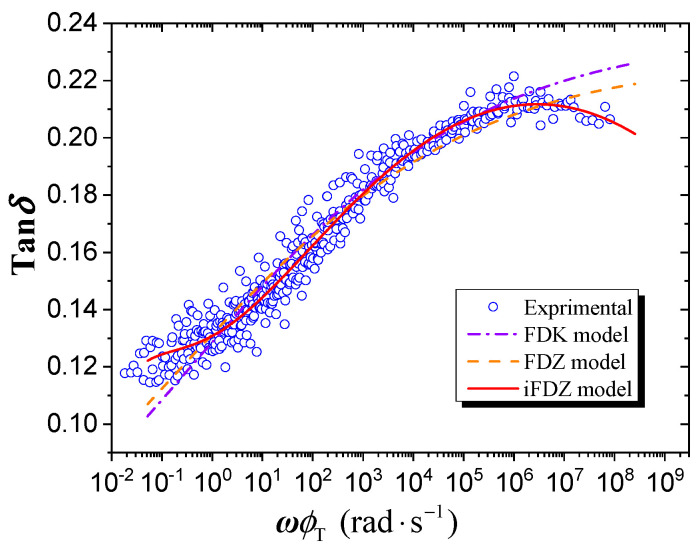
Comparisons of the fractional-order differential models and experimental results.

The Maxwell model, Kelvin-Voigt model, and Zener model are the classical differential rheological models and are binary or ternary models with few parameters. The Maxwell model presents the properties of fluids and the object characterized by the Maxwell model is usually referred to as Maxwell fluid. In contrast, the Kelvin–Voigt model and Zener model present the properties of a rheological solid. The fractional-order derivative model has greater descriptive power than the corresponding integer-order differential model and can degenerate into integer-order differential models as necessary when their fractional order is close to one or zero. For some viscoelasticity materials, the master curves of loss factor and loss modulus are asymmetric. The Maxwell model, Kelvin model, and Zener model and their corresponding fractional-order derivative models cannot effectively describe the asymmetry of dynamic viscoelasticity. The improved fractional order Zener model, due to its two fractional orders, can be used to describe the asymmetry of material dynamic mechanical properties. Although the generalized Kelvin–Voigt and generalized Maxwell model can also represent dynamic viscoelasticity, they have too many parameters [38]. In this research, the fractional-order differential Kelvin–Voigt (FDK) model, the fractional-order differential Zener (FDZ) model and the improved fractional-order differential Zener (iFDZ) model were introduced to approximate and analyze the loss factor master curve of silicone rubber. The results are shown in Figure 12, and the model parameters are listed in Table 3. In the table, *R*^2^ represents the coefficient of determination, and *SD* represents the standard deviation.

In the FDK model, FDZ model, and iFDZ model, *E*_1_, *E*_2_, and *E*_3_ are the elastic moduli in the models. Generally, the higher the crosslinking density of rubber, the greater they are. *τ* means the average relaxation time; here, it refers to the time required for the silicone rubber material to transition from an equilibrium state through polymer motion to a new equilibrium state that is suitable for the external field.

As shown in the figure and table, it can be seen that the FDK model can describe the trend of the master curve of the loss factor of silicone rubber material in the wide frequency range, to a certain extent, but slightly worse so than the FDZ model. In contrast, the iFDZ model closely resembles the asymmetric master curve of the loss factor of silicone rubber and is obviously superior to the FDK model and FDZ model based on the value of *R*^2^ and *SD* in Table 3.

**Figure 13:**
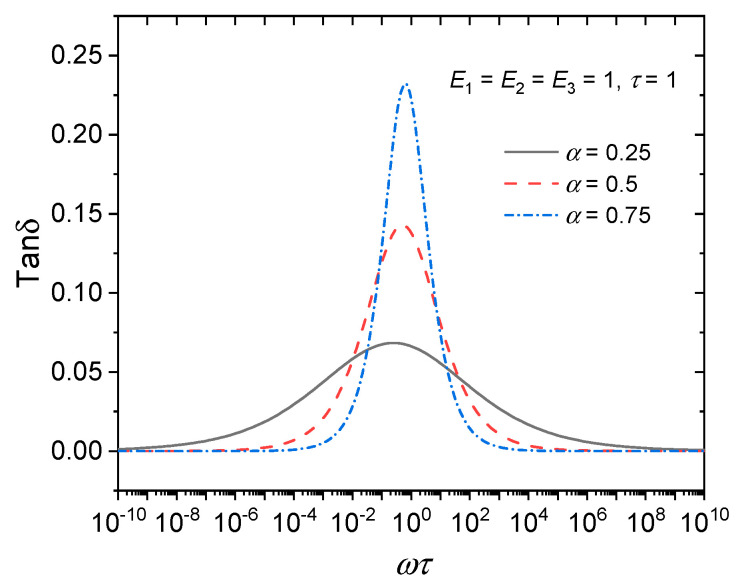
Loss factor curves of the FDZ model.

**Figure 14:**
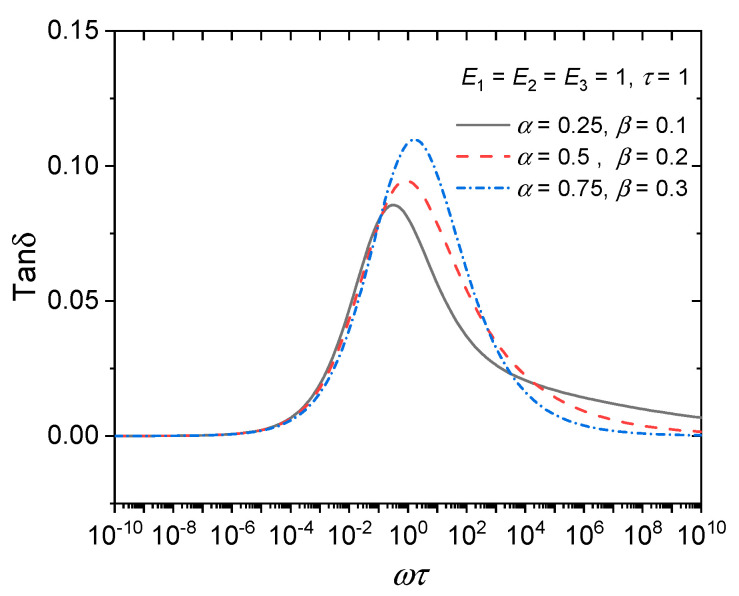
Loss factor curves of the iFDZ model.

According to the constitutive equations of the aforementioned FDK model, FDZ model, and iFDZ model, it can be seen that the FDK model and the FDZ model contain only one fractional order, *α*, which only describes a symmetric loss factor curve, while the iFDZ model can describe a dynamic viscoelastic curve with asymmetry because it contains two fractional orders, *α* and *β*, when *α* and *β* are not equal. If we let *E*_1_ = *E*_2_ = *E*_3_ = 1 and *τ* = 1, and let *α* = 0.25, 0.5, and 0.75, the loss factor curve is plotted as shown in Figure 13 and Figure 14, demonstrating the above conclusions.

Although the iFDZ model contains six material parameters, its parameters are not too numerous compared to many rheological models. Additionally, due to its two fractional orders, α and β, it can describe the asymmetry of dynamic viscoelasticity of materials, which indicates that it is a powerful tool to characterize the asymmetric dynamic viscoelasticity of rheological materials. Noteworthily, the material’s structure and composition may have an impact on the symmetry of dynamic viscoelasticity of materials. In general, when the molecular structure is homogeneous, the curve is symmetric; when the material’s structure is a blend system or heterogeneous, as in microheterogeneous systems, the dynamic viscoelastic curves are asymmetric. For the silicone rubber in this study, asymmetry is related to its vulcanization and fillers [39].

## 5. Conclusions

In this work, the dynamic mechanical thermal analyzer was used to test the frequency spectrum scan of silicone rubber material in the range of different temperatures (−35 °C to 60 °C) levels, and the test showed that the dynamic viscoelastic properties of silicone rubber have obvious temperature–frequency dependence. The dynamic viscoelastic test curves at different temperatures can be shifted along the logarithmic frequency coordinate axis to construct smooth master curves, covering a frequency range of 10 decades, which indicates the thermorheological simplicity and frequency temperature equivalence of the silicone rubber material in the experimental temperature range. Furthermore, the van Gurp–Palmen plot and Cole–Cole plot for the test data at various temperatures merge into a common curve, verifying the material’s thermorheological simplicity on a macro level. In addition, the asymmetric loss factor master curve was approximated by the FDK model, the FDZ model, and the iFDZ model. The results showed that the iFDZ model is in good agreement with the experimental master curve at the angular frequency spans from 10^−2^~10^8^ rad/s, indicating that this model is suitable for describing the asymmetry of dynamic viscoelastic properties of silicone rubber.

## Figures and Tables

**Table 1:** Temperature shift factors at various temperatures (*T*_r_: 20 °C).

**T/°C**	−35	−30	−25	−20	−15	−10	−5	0	5	10
log*ϕ*_T_	5.27	4.48	3.89	3.35	2.84	2.36	1.74	1.43	1.00	0.63
**T/°C**	15	20	25	30	35	40	45	50	55	60
log*ϕ*_T_	0.30	0	−0.34	−0.44	−0.64	−0.84	−1.11	−1.28	−1.49	−1.66

**Table 2:** Parameters *C*_1_ and *C*_2_ of WLF equation at various reference temperatures.

*T*_r_ (K)	*C*_1_	*C*_2_ (K)	*C*_1_ × *C*_2_ (K)	*C*_1_/*C*_2_ (K^−1^)	*R* ^2^	*T*_r_ (K)	*C*_1_	*C*_2_ (K)	*C*_1_ × *C*_2_ (K)	*C*_1_/*C*_2_ (K^−1^)	*R* ^2^
238.15	12.98	82.55	1071.50	0.16	0.999	288.15	7.40	123.20	912.17	0.06	0.9984
243.15	12.27	89.38	1096.70	0.14	0.9988	293.15	7.22	129.10	931.97	0.06	0.9982
248.15	11.48	90.57	1039.74	0.13	0.999	298.15	6.97	132.90	926.58	0.05	0.9957
253.15	11.32	98.96	1120.23	0.11	0.9985	303.15	7.14	146.00	1042.73	0.05	0.999
258.15	11.25	109.80	1235.25	0.10	0.998	308.15	6.75	149.70	1010.03	0.05	0.9987
263.15	11.07	119.20	1319.54	0.09	0.9978	313.15	6.33	152.30	963.60	0.04	0.9985
268.15	8.74	103.20	902.38	0.08	0.9986	318.15	6.40	159.90	1023.20	0.04	0.999
273.15	9.34	119.80	1118.45	0.08	0.9989	323.15	6.04	162.90	983.59	0.04	0.9989
278.15	8.36	117.50	982.65	0.07	0.999	328.15	6.02	169.70	1020.92	0.04	0.999
283.15	7.75	119.00	922.49	0.07	0.9986	333.15	5.86	174.90	1024.91	0.03	0.999

**Table 3:** The material parameters of the fractional-order differential models.

Model	*E*_1_ (MPa)	*E*_2_ (MPa)	*E*_3_ (MPa)	*α*	*β*	*τ* (s)	*R* ^2^	*SD*
FDK	7.19	8.49	/	0.15	/	1.25	0.93	0.0079
FDZ	0.10	821.10	0.15	0.14	/	0.57	0.94	0.0073
iFDZ	0.65	55.21	0.22	0.16	0.66	179.70	0.97	0.0053

## Data Availability

The data presented in this study are available on request from the corresponding author.
